# Measles transmission from an anthroposophic community to the general population, Germany 2008

**DOI:** 10.1186/1471-2458-11-474

**Published:** 2011-06-15

**Authors:** Maria Wadl, Anette Siedler, Wolfgang Krämer, Maria E Haindl, Stephan Gebrande, Irene Krenn-Lanzl, Annette Mankertz, Wolfgang Hautmann

**Affiliations:** 1 Department for Infectious Disease Epidemiology, Robert Koch Institute, Berlin, Germany; 2 Local Health Authority, Berchtesgaden, Germany; 3 Local Health Authority, Traunstein, Germany; 4 Local Health Authority, Rosenheim, Germany; 5 Local Health Authority, Mühldorf am Inn, Germany; 6 National Reference Centre for Measles, Mumps and Rubella, Robert Koch- Institute, Berlin, Germany; 7 Bavarian Health and Food Safety Authority, Department for Epidemiology, Oberschleissheim, Germany

## Abstract

**Background:**

In Germany, measles vaccination coverage with two doses is not yet sufficient to prevent regional outbreaks. Among the 16 German federal states, vaccination coverage was lowest in Bavaria with 85% in 2008. From March to mid-April 2008, four neighbouring Bavarian counties reported 55 measles-cases mostly linked to an ongoing measles outbreak in an anthroposophic school in Austria. We investigated this outbreak to guide future public health action.

**Methods:**

We applied the German national case-definition for measles and collected data using the national surveillance system and a questionnaire. Measles cases with disease onset a maximum of 18 days apart and spatial contact (e.g. same household, same school) were summed up in clusters. Two different interventions, which were implemented in schools and kindergartens in Bavaria, were compared by their impact on the size and duration of measles clusters. Susceptible persons were excluded from schools or kindergartens either with the first (intervention A) or second (intervention B) measles case occurring in the respective institution.

**Results:**

Among the 217 Bavarian measles cases identified from March-July 2008, 28 (13%) cases were attendees of the anthroposophic school in Austria. In total, vaccination status was known in 161 (74%) cases and 156 (97%) of them were not vaccinated. The main factor for non-vaccination was "fear of vaccine-related adverse events" (33%). Twenty-nine (18%) of 161 cases suffered complications. Exclusively genotype D5 was detected. Overall, 184 cases could be epidemiologically grouped into 59 clusters. Of those, 41 clusters could be linked to households and 13 to schools or kindergartens. The effect of intervention A and B was analysed in 10 school or kindergarten clusters. Depending on the respective intervention A or B, the median number of cases per cluster was 3 versus 13 (p = 0.05), and the median duration of a cluster was 3 versus 26 days (p = 0.13).

**Conclusions:**

Introduction of measles virus into a pocket of susceptible persons (e.g. vaccination opponents or sceptics) may lead to large outbreaks in the general population, if the general population's vaccination coverage is below the WHO recommended level. Education on the safety of measles vaccine needs to be strengthened to increase measles vaccination coverage. Early intervention may limit spread in schools or kindergartens. Suspected measles has to be reported immediately to the local health authorities in order to allow intervention as early as possible.

## Background

Infection with measles virus is potentially severe and the leading cause of vaccine-preventable childhood mortality worldwide. Humans are the only reservoir, and treatment is only symptomatic. The incubation period is about 10 days, and ranges between 7 and 18 days from exposure to onset of fever; rarely, it is as long as 19-21 days. The period of contagiousness is usually 4 days before and after onset of rash [1]. Results from recent outbreak investigations in Austria, Germany and Switzerland showed, that measles disease mostly affected persons 10 years and older [2-4].

The WHO aims to eliminate measles in the WHO European region by 2015 [5]. One indicator for measles elimination would be an incidence of < 0.1 cases per 100,000 inhabitants.

In Germany, measles is a notifiable disease. The median annual incidence between 2003 and 2007 was 0.9 cases per 100,000 inhabitants, and the lowest incidence in this time period was reported in 2004 with 0.2/100,000. Incidence rose to 2.8/100,000 in 2006, due to a large outbreak with 1749 notified cases [6].

High protection rates are achieved after two doses of measles vaccine. Vaccine effectiveness after two doses of measles vaccine in two outbreak settings was 95% and 99% [4,7]. In order to prevent outbreaks, a measles vaccine coverage for two doses of 95% is needed [8]. The German Standing Committee on Vaccination (STIKO) recommends the first dose of measles vaccine to all children at 11-14 months of age and a second dose at 15-23 months of age.

Coverage can vary by subgroups for medical, religious, philosophical or personal reasons [9].

In 2008, vaccination coverage of first graders for two doses of measles vaccine was 89% (range: 85-94%) in Germany. Among the 16 federal states, the lowest vaccination coverage (85%) was reported in Bavaria [10].

### Outbreak background

At the end of March 2008, physicians notified 15 persons with measles disease residing in two neighbouring Bavarian counties to the respective local health authorities. Of those, 14 attended an Austrian anthroposophic school with an ongoing measles outbreak since the beginning of March [3,11]. The affected German counties share a border with Austria, hence regular school attendance in areas across the border is not unusual. The Austrian outbreak investigation team found a 34% (101 of 294) coverage with at least one dose of measles vaccine among students attending the anthroposophic school in Salzburg city [3]. Other investigations in European countries had already yielded, that immunisation against measles, mumps, and rubella (MMR) was lower in students attending anthroposophic schools than in those attending non-anthroposophic schools [12][13]. Hence, these students may form a pocket of susceptible persons.

Until mid-April 2008, the number of measles cases possibly related to the outbreak in the Austrian school increased to 55, affecting the population of four neighbouring Bavarian counties. Between 2001 and 2007, a median of seven measles cases per year had been notified in the four counties. The average vaccination coverage of first graders for two doses of measles vaccine in the four counties was 69% (range 64-72%) in 2006/2007. We report about the outbreak investigation, which was initiated to describe the public health implications of the measles outbreak in Bavaria, to explore the reasons for non-vaccination and to compare the effect of implemented control measures in order to guide future public health action.

## Methods

### Data collection and descriptive analysis

Measles is a notifiable disease in Germany. Physicians must report any suspected case, clinical cases of and deaths from measles within 24 hours to the responsible local health authority. Heads of laboratories are obliged to report any direct or indirect evidence of measles virus --- if the evidence suggests an acute infection - within 24 hours to the responsible local health authority. The German local health authorities routinely collect case based data, e.g. on demographics, clinical symptoms, hospitalisation, vaccination status, travel history and laboratory testing. Contact tracing is also performed as a standard procedure. Local health authorities of two Bavarian counties accepted self reporting of the vaccination status by patients while the other two Bavarian counties requested verification by physicians or health inspectors. In this outbreak investigation, we defined a case as any resident of the four affected Bavarian counties (Berchtesgadener Land, Rosenheim, Traunstein, Mühldorf am Inn), diagnosed with clinical measles and disease onset between February 1 and August 30, 2008. Clinical measles was defined as a generalised maculopapular rash persisting for at least 3 days, fever ≥ 38.5°C, and at least one of the four following symptoms: conjunctivitis, cough, runny nose or Koplik's spots. Cases were further categorized as having a link with the Austrian outbreak or not. We considered "attending school in Austria", "visiting Salzburg city" or "having contact with a measles case from Salzburg city" during the incubation period as potential links to the Austrian outbreak.

Cases were notified by physicians and laboratories to the respective local health authority (LHA) which entered the information into the electronic surveillance system. Additionally, the LHA collected data on reasons for non-immunization and complications from all notified cases through a questionnaire administered by telephone or post. If patients were under age 16, parents were queried. Study participation was voluntary. We assumed implicit informed consent when a completed questionnaire was returned. Information on non-responders was available.

We used EpiData software version 3 (EpiData Association, Odense, Denmark) for double data entry, and Stata (Stata Statistical Software: Release 10, Texas, USA) for descriptive analysis.

### Laboratory analysis

Laboratory confirmation using serology was performed by regional laboratories. The National Reference Centre for Measles, Mumps and Rubella in Berlin (NRC) used the Enzygnost Anti-Measles Virus IgM ELISA (Siemens, Germany) for the detection of anti-measles IgM in serum. A subset of samples was genotyped by the NRC according to the WHO recommendation for measles virus [14]. As described in Santibanez et al., 2002 [15], viral RNA was extracted from urine or throat swab specimens and reversely transcribed into cDNA. A C-terminal 450 nt fragment corresponding to the hypervariable 150 aa of the N-protein of measles virus was amplified by PCR and subsequently sequenced. The sequences were phylogenetically analysed.

### Clusters and applied control measures

Cases with disease onset a maximum of 18 days apart and spatial contact (e.g. same household, same school) were grouped into clusters.

In Germany, the LHAs are responsible for the implementation of control measures. In order to assess the effectiveness of implemented control measures, the Robert Koch Institute interviewed the LHAs by telephone. Our interviews of the local health authorities revealed that they applied two different control measures in schools and kindergartens (preschool) during the outbreak: the trigger for exclusion of non-immune persons from the respective school or kindergarten for 14 days after the last contact to an infectious case was either the notification of at least one measles case (intervention A) or at least two measles cases (intervention B) in the respective school or kindergarten. Immune persons were those with at least one documented vaccination against measles a minimum of three weeks before disease onset, immunity confirmed by serology or anamnestic measles. Intervention A and B were implemented compulsory. Compliance was not controlled for. Vaccination cards in schools and kindergartens were checked by staff from the LHAs in three of the affected counties, and by staff from the respective school or kindergarten in the fourth county.

Attack rates were calculated and negative binomial regression was used to estimate the average attack rate ratio with 95% confidence intervals (95% CI) according to the implemented measure. Teachers and staff of schools and kindergartens were not included in the denominators of the specific institutions.

In order to analyse the effect of the respective intervention we excluded the case with the first disease onset from all clusters in which intervention A was applied and the two cases with the first disease onsets from all clusters where intervention B was used. The duration of clusters by intervention was calculated starting with the onset of the respective intervention. The school cluster in the anthroposophic school in Salzburg city (since Austrian health authorities were responsible for the intervention) and clusters in kindergartens and schools with all cases occurring on the same day (0 days apart, mode of intervention did not differ) were excluded from analysis.

We compared the median number of cases per cluster and the median duration of clusters in days according to the implemented intervention using the Mann-Whitney-U-Test. Using a Poisson regression model we estimated the average decline of cases per 7-day interval. We categorized the days of disease onsets in 7-day intervals, starting with the onset of the second case in clusters with intervention A and of the third case in clusters with intervention B as day 1.

## Results

### Outbreak description

**Figure 1:**
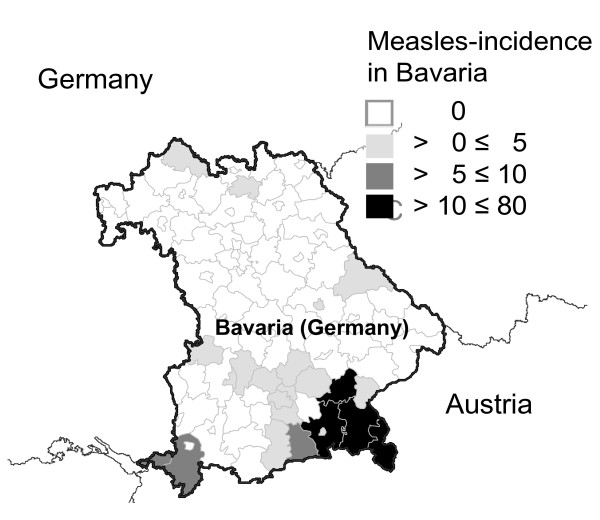
**Measles incidence (cases per 100,000 population) by county**. Measles outbreak in four Bavarian counties, Germany, March-July 2008. Counties with highest incidences were included in this study (shaded in black).

From 14 March to 15 July (calendar weeks 11-29) 2008, we identified 217 measles cases in the four counties. No case was found in teachers or staff of schools and kindergartens. In the outbreak period, the four counties had an average measles incidence of 32 cases per 100,000 population (range: 12-71) which was the highest among all Bavarian counties (overall: 2 cases per 100,000 population, Figure 1). Six weeks before and after the outbreak period, no other measles case was reported within the four counties. For the 215 measles cases with available information on date of onset and date of reporting the mean reporting delay was 6 days (range: 0-28 days); the reporting delay did not differ among the four counties.

**Figure 2:**
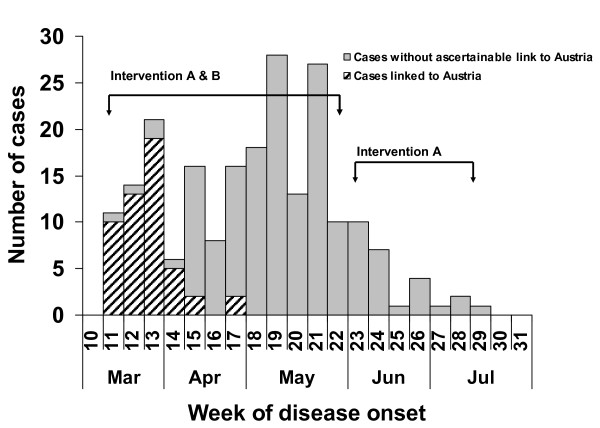
**Measles cases by week of onset of disease**. Measles outbreak in four Bavarian counties, Germany, March-July 2008 (N = 217).

Fifty-one cases (24%) could be linked to the ongoing measles outbreak in Austria. Among those, 28 attended the same anthroposophic school in Salzburg city. All linked cases occurred in the first seven weeks of the outbreak. Case numbers peaked in May, with 28 and 27 cases respectively in week 19 and 21. After that, case numbers decreased continuously (Figure 2).

**Figure 3:**
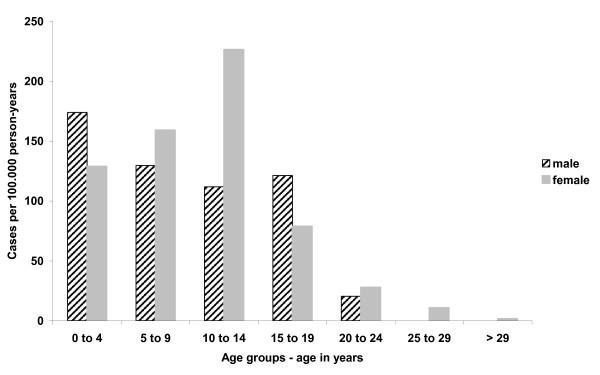
**Measles incidence rate (cases per 100,000 person-years) by sex and age group (age in years)**. Measles outbreak in four Bavarian counties, Germany, March-July 2008 (N = 217).

The median age of cases was 11 years (range: 0-57), 47% were male. The incidence rate was highest in persons aged 10-14 years (170 cases/100,000 person-years), followed by those 0-4 years (150/100,000) and 5-9 years (140/100,000) of age (Figure 3).

Overall, 25 of 217 (11%) cases were hospitalised for a median of four days (range: 1-13 days). The hospitalised patients had a median age of 16 years in contrast to 10 years for the non-hospitalized group (p = 0.001).

Of the 217 cases, 161 (74%) responded to the questionnaire. The non-responder analysis revealed geographic differences: while one county managed a response of 100%, the others achieved 56%, 60% and 62% (P < 0.001). The non-responders did not differ regarding age, sex, vaccination status or epidemiological link to Austria.

Twenty-nine cases (18%) of 161 developed complications; most frequently, otitis media was reported (n = 14). A 13-year-old girl, who was hospitalised for five days, suffered from seizures (Table 1).

**Table 1:** Complications of measles.

Disease complications*	Number	%
Otitis media	14	8.7
Disturbance of consciousness	9	5.6
Diarrhoea	9	5.6
Disorientation	8	5.0
Mood shifts	8	5.0
Pneumonia	6	3.7
Seizures	1	0.6

Measles outbreak in four Bavarian counties, Germany (March to July, 2008) (n = 161/217).

* multiple answers possible

Overall, 156 (97%) of 161 measles cases filling in the questionnaire were unvaccinated. Four persons had received one measles vaccine dose, one patient two doses. All vaccinations had been applied at least one year before symptom onset.

The main reasons for not being vaccinated against measles were "fear of vaccine-related adverse events" (33%), "opposing measles vaccination in general" (30%) and the opinion "measles is not a severe disease" (18%) (Table 2).

**Table 2:** Reason for non-immunization among measles cases.

Reasons for non-immunization*	Number	%
"Being afraid of vaccine side-effects"	52	33.3
"Opposing measles vaccination in general"	47	30.1
"Believing, measles is not a severe disease"	28	17.9
"Doctor advised against vaccination"	19	12.2
"Vaccination missed"	15	9.6
"Vaccination forgotten"	10	6.4
"Vaccination not offered"	7	4.5

Measles outbreak in four Bavarian counties. Germany. March-July 2008 (n = 156/217)

* multiple answers possible; closed questions

### Laboratory analysis

In total, 54 (25%) of 217 cases were laboratory confirmed by serology. Two of the five vaccinated persons, including the patient vaccinated twice were among the confirmed cases. Virus isolates from 28 cases were genotyped. In two cases, sequencing of the viral genome was not successful, while in the remaining 26 cases, identical sequences revealing genotype D5 were detected. This virus variant was found in all four counties and included patients with and those without links to Austria.

### Clusters and effect of differently applied control measures in schools and kindergartens

A total of 184 (85%) cases could be epidemiologically grouped to 59 clusters. These could be linked to 41 households, 13 schools or kindergartens, four hospitals or medical practices and one firemen's festival (Table 3). A total of 32 cases were linked to the use of a school bus which carried children and teenagers to and from three of the affected schools. However, all cases linked with the school bus were included in the respective school clusters.

**Table 3:** Description of measles clusters by type and size.

	Clusters (n = 59)		Cases within clusters*		
		
Type of cluster	*N*	*%*	*N*	*Median*	*Range*
Household	41	69	112	3	2-8
School or kindergarten	13	22	163	9	2-28
Hospital or medical practice	4	7	12	4	2-7
Firemen's festival	1	2	7	7	

* multiple cases per type of cluster possible

Overall, we included 10 clusters, three in kindergartens and seven in schools, with 85 cases altogether from all four counties for the comparison of the effect of intervention A and B (Table 4). Two schools had anthroposophic teaching methods. Local health authorities recommended intervention A during six clusters (28 cases) and intervention B during four clusters (57 cases), based on their own decision.

**Table 4:** Description of measles clusters in schools and kindergartens.

Type of Institution	Anthro-posophic	Week of disease onset of 1^st ^case	Number of cases per cluster (N)	N, after start of respective intervention	Duration of cluster in days (D)	D, after start of respective intervention	Total number of children in institution (I)	Attack rates* in %
***Intervention A***								
School	yes	11	2	1	4	0	25	8.0
School	yes	15	6	5	21	20	600	1.0
Kindergarten	no	17	2	1	10	0	99	2.0
Kindergarten	no	17	8	7	12	5	50	16.0
School	no	23	8	7	37	36	530	1.5
School	no	24	2	1	8	0	170	1.2
					
			**Total **= 28	**Median **= 3		**Median **= 3	**Total **= 1474	**Mean **= 1.9

***Intervention B***								
Kindergarten	no	17	15	13	24	23	72	20.8
School	no	18	4	2	10	3	364	1.1
School	no	18	15	13	31	28	507	3.0
School	no	19	23	21	67	66	787	2.9
					
			**Total **= 57	**Median **= 13		**Median **= 26	**Total **= 1730	**Mean **= 3.3

* The attack rates should be interpreted with caution since the population at risk (number of susceptible contacts per school or kindergarten) was not available.

Three counties implemented solely intervention A. The fourth county used intervention B until calendar week 22 (12 weeks) and intervention A since calendar week 23 (7 weeks, Figure 2). The county using intervention B informed as early as the occurrence of the first incident measles case in a kindergarten or school all contact persons about the incident measles case and the necessity of protection by vaccination.

Both, the attack rates as well as the average attack rate ratios were calculated but should be interpreted with caution since the population at risk (number of susceptible contacts per school or kindergarten) was not available: The mean attack rate in institutions was 2.7%. Stratification by intervention led to a mean attack rate of 1.9% (range: 1-16%) if intervention A was used and 3.3% (range: 1-21%) if intervention B was applied. Average attack rate ratios in institutions where intervention A was implemented were 1.6 times lower (95% CI: 0.4-6.1) than those of institutions where intervention B was applied.

For the following analyses - as described in the methods section - we neither counted the first case of clusters A nor the first two cases of clusters B; furthermore, the calculation of the cluster duration in days started with the first day of the respective intervention.

Clusters in kindergartens and schools had a median number of 3 (intervention A; range: 1-7) versus 13 (intervention B; range: 2-21) cases (p = 0.05), and lasted a median of 3 (intervention A; range: 0-36) versus 26 (intervention B; range: 3-66) days (p = 0.13).

**Figure 4:**
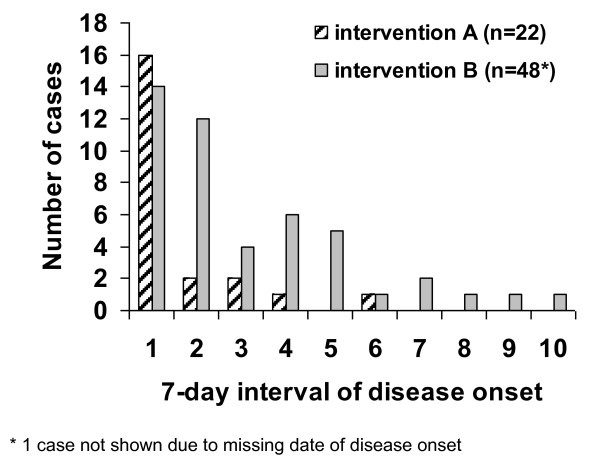
**Number of cases and duration of clusters in kindergartens and schools per 7-day intervals and by implemented intervention**. For this analysis we neither counted the first case of clusters A nor the first two cases of clusters B. Considering the latter, the day of disease onset of the second (cluster A) and third (cluster B) case, respectively, in each school and kindergarten cluster is set as 1; the days of disease onsets are categorized in 7-day intervals (n = 70).

The estimated mean decrease of cases per 7-day interval was 58% using intervention A and 29% intervention B, indicating that the average number of cases per 7-day interval decreased 1.7 times (95% confidence interval: 1.1-2.7; p = 0.03) slower using intervention B compared to intervention A (Figure 4).

## Discussion

Our investigation showed, that introduction of measles virus into a pocket of susceptible persons like the students of the anthroposophic school in Salzburg city provoked an outbreak and was followed by further spread to the general population with a vaccination coverage below the WHO recommended level. Furthermore, persons in schools and kindergartens, who were susceptible for measles, should have stayed home with the first known measles case in the respective institution. In order to start control measures as early as possible any suspected measles case has to be reported without delay.

In this outbreak investigation, most cases were not vaccinated against measles. The reasons for not being vaccinated among cases indicate that parents lack knowledge about the true risks associated with vaccination or with non-vaccination, as described in other European studies [9,16,17]. Internationally available measles vaccines are regarded as safe and effective [18], and only minor adverse effects have been reported with measles vaccine [19,20].

Studies have shown that an increase in knowledge and a change in behaviours can be achieved through awareness campaigns [21,22]. Health professionals should use all opportunities (e.g. contact with patients), both to inform about the highly protective effect of measles vaccinations and to offer measles vaccination to persons, who are not yet immunized.

The relatively high median age at infection was probably the consequence of a partially immunized population, which leads to a reduced rate of virus circulation and thus raises the age at which persons get infected. Higher age of measles cases may lead to more serious diseases [23] which is reflected in a higher proportion of hospitalisations among older cases than younger cases in our study. A considerable percentage of cases (18%) in our study reported complications, including one case that had seizures. This indicates that measles is not a mild disease.

The measles virus genotype D5 detected in this outbreak was identical to the one causing an outbreak in Austria at the same time. This supports the link to Austria, which was also found in the epidemiological investigations.

Schmid and colleagues reported that the primary case of the outbreak in Austria came from Switzerland; the genotype circulating in Austria was the same as in our findings and was indistinguishable from the outbreak strain in Switzerland [3].

The high number of clusters in households and institutions such as schools or kindergartens underlines the infectiousness of the measles virus. The compared attack rates should be interpreted with caution since the number of persons who were already immune against measles was not available for the population at risk in the respective institutions. Therefore, we can assume that the calculated attack rates are rather underestimates.

The differently applied control measures in schools and kindergartens offered us a unique opportunity to study the effect of early interventions in this measles outbreak. The mean number of cases and duration per cluster indicated that exclusion of susceptible persons from schools and kindergartens starting with the first incident measles case (intervention A) might be more effective than exclusion of susceptible persons after the second case (intervention B). However, only the comparison of the median number of cases per cluster showed borderline statistically significant results, if accepting an alpha-error-level of 0.05. We did not use the calculated attack rates and mean attack rate ratios as basis to assess the effectiveness of the respective interventions, since the population at risk (number of susceptible contacts per school or kindergarten) was not sufficiently available. Intervention A is also recommended by a guideline from the German federal state of Lower Saxony [24]. Measles have a high basic reproduction number (R_0 _12-18) and contacts among school and kindergarten attendees are frequent. Therefore, it is biologically plausible that early intervention will be more successful, which is supported by our observations. To start intervention as early as possible, suspected measles cases have to be immediately notified by physicians to the health authorities. The detected reporting delay might have influenced the outcome of the control measures.

This study had some limitations. First, case finding was based on the reporting of primary care physicians to the LHA which possibly underestimated the extent of the outbreak. Active case finding was limited to contacts of notified measles cases.

Second, interviews were either done by telephone or by self-administered questionnaires, so interviewer bias could have been present. We tried to counter that by the use of a standardized questionnaire.

Third, information on the population at risk (number of susceptible contacts per school or kindergarten) was not sufficiently available. Therefore, attack rates were compared among all children and/or teenagers attending the affected schools and kindergartens.

Last, the duration of the school and kindergarten clusters may also depend on the number of cases and hence of attendees in each institution.

## Conclusions

This outbreak investigation shows that once the measles virus has found its way into a low-immunized population like an anthroposophic community, the general population --- if having vaccination coverage below the WHO recommended level, such as in Bavaria - is at risk of measles outbreaks. Furthermore, early isolation of non-immune persons might limit the size and duration of clusters in schools and kindergartens effectively.

Uncertainty about the safety of measles vaccine and underestimation of the risks of the disease has contributed to the low coverage of measles vaccination. In order to improve the overall vaccination coverage, we recommend awareness campaigns targeting health professionals and parents. Physicians and parents should be informed about the true risks associated with vaccination compared with non-vaccination. Moreover, health professionals have to be convinced that immediate notification of suspected measles cases to the health authorities is important for timely infection control measures. The early intervention method should be considered in future outbreaks, particularly if a population with moderate vaccination coverage is affected.

## Competing interests

The authors declare that they have no competing interests.

## Authors' contributions

MW analysed and interpreted the data and drafted the manuscript. AS and WH made substantial contributions to conception and design, analysis and interpretation of the data and coordinated the study. WK, MEH, SG and IKL made substantial contributions to acquisition of data. AM performed laboratory analyses. All authors have been involved in drafting the manuscript and revising it critically. All authors read and approved the final manuscript.

## Pre-publication history

The pre-publication history for this paper can be accessed here:

http://www.biomedcentral.com/1471-2458/11/474/prepub
